# PRV gD-Based DNA Vaccine Candidates Adjuvanted with cGAS, UniSTING, or IFN-α Enhance Protective Immunity

**DOI:** 10.3390/pathogens14101026

**Published:** 2025-10-11

**Authors:** Xinqi Shi, Shibo Su, Yongbo Yang, Liang Meng, Wei Yang, Xinyu Qi, Xuyan Xiang, Yandong Tang, Xuehui Cai, Haiwei Wang, Tongqing An, Fandan Meng

**Affiliations:** 1 State Key Laboratory for Animal Disease Control and Prevention, Harbin Veterinary Research Institute, Chinese Academy of Agricultural Sciences, 150069 Harbin, China; xinqishi2727@163.com (X.S.); sushibo@caas.cn (S.S.); yangyongbo@caas.cn (Y.Y.); mengl98@163.com (L.M.); qxy4033@163.com (X.Q.); xyxiang1218@163.com (X.X.); tangyandong@caas.cn (Y.T.); caixuehui@caas.cn (X.C.); wanghaiwei@caas.cn (H.W.); 2 College of Veterinary Medicine, Northeast Agricultural University, 150030 Harbin, China; yangwei0727@hotmail.com; 3 Heilongjiang Research Center for Veterinary Biopharmaceutical Technology, Harbin Veterinary Research Institute, Chinese Academy of Agricultural Sciences, 150069 Harbin, China; 4 Heilongjiang Provincial Key Laboratory of Veterinary Immunology, Harbin Veterinary Research Institute, Chinese Academy of Agricultural Sciences, 150069 Harbin, China

**Keywords:** PRV, adjuvant, DNA vaccine

## Abstract

Pseudorabies virus (PRV), a major swine pathogen, causes severe neurological, respiratory, and reproductive disorders, resulting in substantial economic losses to the global swine industry. Previous studies have shown that the gD glycoprotein of PRV has an effective protective effect. In this study, we constructed a plasmid DNA vaccine (pVAX1-GD-Fc) encoding a gD protein fused with pig IgG Fc and evaluated the adjuvant effects of porcine cGAS, the universal STING complex mimic (UniSTING), or IFN-α in mice. The mice were immunized three times (days 0, 14, and 21) with pVAX1-GD-Fc in the presence or absence of an adjuvant, followed by lethal challenge with PRV-HLJ8 3 days after the final immunization. The results revealed that the pVAX1-GD-Fc group exhibited 20% mortality (1/5 mice) on day 7 postchallenge, and all adjuvanted groups achieved 100% survival during the 14-day observation period. Flow cytometric analysis of splenocytes one week after the second immunization revealed significantly greater CD8+ T cell proportions in the adjuvant groups than in both the mock and pVAX1-GD-Fc-only control groups (*p* < 0.01). Furthermore, T cell proliferation assays demonstrated a significantly increased stimulation index in the adjuvant-treated mice, confirming enhanced cellular immunity. These findings demonstrate that cGAS, UniSTING, and IFN-α can serve as effective vaccine adjuvants to rapidly enhance cellular immune responses to PRV, highlighting their potential application in veterinary vaccines.

## 1. Introduction

Porcine pseudorabies is a highly contagious disease caused by pseudorabies virus (PRV), which is endemic worldwide and affects susceptible animals such as pigs, cattle, and sheep [1]. In piglets under one month of age, infection results in a mortality rate approaching 100%, with common signs including diarrhea, fever, and ataxia [2]. PRV is an enveloped double-stranded DNA virus belonging to the *Herpesviridae* family, specifically the *Alphaherpesvirinae* subfamily [3]. The viral envelope of PRV expresses 11 glycoproteins, namely, gB, gC, gD, gE, gG, gH, gI, gK, gL, gM, and gN [4]. The gD protein, a crucial ligand for viral entry into host cells, binds to receptors on the cell surface. As a primary glycoprotein of PRV, gD stimulates the host to produce neutralizing antibodies against the virus [5]. PRV is globally prevalent and continues to inflict significant economic losses on the global pig farming industry. While several countries in North America and Europe have declared its eradication, the virus continues to be widespread in China [6]. PRV has been prevalent in China since the 1950s. Although the PRV Bartha-K61 vaccine strain was subsequently introduced for prevention and control and has achieved significant effectiveness [7], various PRV strains have spread across the country since 2011 and remain endemic in China [8,9]. In addition, the seroprevalence of PRV gE antibodies in pig farms across 14 provinces in China from 2017 to 2021 revealed a declining trend in PRV gE seropositivity over time [10]. However, PRV often coexists with multiple other pathogens, including porcine circovirus (PCV), porcine reproductive and respiratory syndrome virus (PRRSV), and classical swine fever virus (CSFV) [11]. Consequently, the development of combination vaccines capable of simultaneously preventing infection by multiple pathogens has become a critical direction in current swine vaccine research. Examples include trivalent vaccines targeting PRV, PCV, and CSFV, for which nucleic acid vaccines represent a highly promising platform owing to their suitability for multivalent formulation.

Currently, research on PRV vaccines focuses mainly on inactivated vaccines, live gene-deleted vaccines, live attenuated recombinant vaccines, DNA vaccines and subunit vaccines. In comparison, live gene-deleted vaccines are most commonly used in clinical practice to protect pigs against PRV infection. Inactivated vaccines exhibit a better safety profile and do not pose a risk of virulence reversion, but their efficacy is generally inferior to that of live vaccines [12]. Subunit vaccines and nucleic acid vaccines have garnered significant attention because of their safety and efficacy profiles in both research and clinical applications. The PRV gD protein subunit vaccine has been demonstrated to elicit robust neutralizing antibody responses in mice and swine, providing effective immunity against PRV challenge [13]. mRNA vaccines and DNA vaccines, as nucleic acid vaccines, hold broad development prospects in the prevention of viral diseases. They demonstrated rapid development and outstanding immunogenicity during the COVID-19 pandemic and can elicit robust cellular and humoral immune responses. However, mRNA vaccines are characterized by poor stability, a reliance on ultracold storage and transportation, and relatively high costs. By contrast, DNA vaccines exhibit weaker immunogenicity, likely because of their low nuclear entry and expression efficiency. Nevertheless, they offer advantages, including low-complexity production, high stability, and the ability to remain stable at room temperature for extended periods [14]. DNA vaccines for veterinary use offer significant advantages in terms of immunogenic efficacy, production costs, and room-temperature storage during transportation. However, compared with live vaccines, nucleic acid vaccines and subunit vaccines exhibit weaker immunogenicity [15].

The cyclic GMP-GAP synthase-stimulator of interferon genes (cGAS-STING) signaling axis, which is involved in various pathological processes, is considered a highly promising research hotspot for vaccine adjuvant development. cGAS can be stimulated and activated by viral DNA or intracellularly accumulated DNA to generate cGAMP, which further activates STING, causing STING to translocate from the endoplasmic reticulum to the Golgi apparatus and activating TBK1 and IRF3. Dimerized IRF3 then translocates into the nucleus, ultimately stimulating the transcription of type I interferon (IFN-I) [16]. IFN-I binds to IFNAR on the cell surface, activating the JAK/STAT pathway and inducing the production of interferon-stimulated factors, thereby exerting antiviral effects [17]. Activation of the cGAS-STING pathway by agonists initiates this signaling cascade, inducing IFN-I production while promoting CD8+ T cell activation and modulating CD4+ T cell differentiation, ultimately potentiating localized antigen-specific immune responses [18]. As a DNA virus, PRV is recognized by cGAS upon entry into cells, activating the cGAS-STING signaling pathway and stimulating the production of IFN-I. However, PRV has also developed multiple strategies to suppress the activation of the cGAS-STING pathway and evade innate immune responses. The PRV UL13 protein can inhibit cGAS-STING-mediated IFN-β production by phosphorylating IRF3 [19]. PRV US2 interacts with the LBD domain of STING and recruits the E3 ubiquitin ligase TRIM21, promoting K48-linked ubiquitination and STING degradation, thereby inhibiting IFN signaling and successfully evading the host antiviral response [20]. These findings also suggest that endogenous cGAS-STING signaling is likely suppressed during PRV infection. Therefore, exogenous agonists, such as cGAS and STING, may compromise the inhibitory effect of PRV on the endogenous cGAS-STING pathway and consequently promote host antiviral responses.

Multiple studies have employed cGAS-STING pathway agonists as vaccine adjuvants to stimulate the activation of this signaling axis, thereby enhancing both innate and adaptive immune responses to improve vaccine protection. STING agonist cyclic dinucleotides (CDNs) as adjuvants enhance the efficacy of a subunit vaccine against Mycobacterium tuberculosis, showing protective efficacy in mice comparable to that of a live attenuated vaccine [21]. However, CDNs face challenges such as high molecular weight, susceptibility to hydrolysis, and suboptimal pharmacokinetic properties. Some small-molecule STING agonists, such as MSA-2, are oral nonnucleotide STING agonists that can effectively stimulate interferon-β secretion in tumors, induce durable tumor regression through antitumor immunity, and have synergistic effects with anti-PD-1 treatment [22]. In addition, incorporation of the STING agonist CF501 into a SARS-CoV-2 vaccine formulation elicited potent long-lasting neutralizing antibody responses in nonhuman primates, whereas downregulation of the cGAS-STING pathway reduced severe lung inflammation induced by SARS-CoV-2 [23].

However, when the cGAS-STING pathway is inhibited, the use of agonists as adjuvants may impair the transduction of downstream signaling pathways. Consequently, several studies have modified the STING protein directly, allowing it to bypass the endogenous pathway and directly activate the cGAS-STING signaling cascade upon administration [24,25,26]. The universal STING complex mimic (UniSTING) can activate downstream STING signaling pathways in various murine and human cells independent of endogenous STING expression [26]. In addition, as a sensor for exogenous viral DNA and self-DNA, cGAS can be activated by either viral or self-derived DNA to stimulate the production of IFN-I. Wild-type cGAS and its variant cGASΔN exhibit different response intensities upon stimulation with self-DNA or viral DNA. In human cells, wild-type cGAS responds more strongly to viral DNA, whereas N-terminal deletions of cGAS result in a stronger response to self-DNA stimulation [27,28]. Currently, few studies have investigated the impact of N-terminal deletion on IFN-I production by porcine cGAS. In addition, recombinant highly glycosylated IFN-α exhibited sustained antiviral activity in vivo and increased neutralizing antibody levels after it was coadministered with an inactivated foot-and-mouth disease vaccine in mice and pigs, indicating that it has a strong adjuvant effect [29].

In this study, UniSTING, cGAS, cGASΔN, and IFN-α were selected as potential adjuvants to determine their ability to increase DNA vaccine immunogenicity through cGAS-STING pathway activation independent of endogenous protein stimulation. We evaluated the ability of the cGAS, cGASΔN, UniSTING, and IFN-α proteins to stimulate IFN-I production and interferon-stimulated gene (ISG) expression in cells. All four candidates effectively inhibited the replication of IFN-sensitive viruses (vesicular stomatitis virus, VSV) and PRV. We subsequently explored the potential of these adjuvants to enhance immunity in vivo by codelivering the PRV GD antigen and adjuvants in the form of a DNA vaccine into mice. After challenge, the vaccine group with added adjuvants demonstrated complete protection of mouse survival. These results also confirm that using the active forms of cGAS, UniSTING, and IFN-α as adjuvants is feasible and may enhance the immune protection of PRV DNA vaccines.

## 2. Materials and Methods

### 2.1. Cells and Viruses

African green monkey kidney (Vero) cells (passage 15), porcine kidney (PK-15) cells (passage 20), and human embryonic kidney (HEK293T) cells (passage 10) were obtained from the American Type Culture Collection (ATCC, Manassas, VA, USA) and maintained in our laboratory. All the cell lines were mycoplasma free and maintained by our laboratory and were cultured in Dulbecco’s modified Eagle’s medium (DMEM; Sigma-Aldrich, St. Louis, MO, USA) supplemented with 10% fetal bovine serum (FBS; Gibco, Grand Island, NY, USA) at 37 °C in an incubator with 5% CO_2_. The PRV HLJ8 strain (GenBank: KT824771.1) was isolated from piglet brain tissue in Heilongjiang Province by our laboratory in 2013 [30]. PRV HLJ8 infection leads to more than 90% lethality in both mice and piglets [31]. Primary porcine alveolar macrophages (PAM) were isolated from the lungs of SPF pigs and were cultured in RPIM 1640 (Sigma-Aldrich, St. Louis, MO, USA) supplemented with 10% FBS at 37 °C in an incubator with 5% CO_2_. PRV HLJ8 [30], PRV-GFP, and VSV-GFP were maintained in our laboratory.

### 2.2. Sequence Design and Plasmid Construction

The cGAS and STING sequences were derived from primary PAM cells. Briefly, primary PAM were stimulated with PRV HLJ8, and the cells were harvested at 4 h and 12 h poststimulation. Total RNA was extracted and reverse transcribed into cDNA, which served as the template for PCR amplification of the full-length cGAS and STING sequences using the primer pairs cGAS-F (5′-ATGGCGGCCCGGCGGGGAAAGTC-3′) and cGAS-R (5′-CCAAAAAACTGGAAATCCATTG-3′) STING-F (5′-ATGCCCTACTCCAGCCTGCATC-3′) and STING-R (5′-GAAGATATCTGAGCGGAGTGG-3′). The tetramerization sequence (Tetrabrachion) with a (G_4_S)_3_-linker sequence (Tebra-GS) and porcine IFN-α (GenBank accession number NM_214393.1) were codon optimized and synthesized by Beijing Liuhe BGI Genetics (Beijing, China). Specific primers were designed to amplify the full-length cGAS (cGAS-F1/R1), cGASΔN (lacking the N-terminal 1–132 amino acids) (cGASΔN-F/R), Tebra-GS (Tebra-F/R), STINGΔN (lacking the N-terminal 1–138 amino acids) (STINGΔN-F/R), and IFN-α sequences (IFN-α-F/R) (primer sequences are listed in Table 1). The pVAX1 and pVAX1-Flag vectors, maintained in our laboratory, were linearized using Nhe I and Hind III restriction enzymes (Thermo Fisher, Waltham, MA, USA). The PCR products were subsequently cloned and inserted into the linearized pVAX1 vector and pVAX1-Flag vectors via homologous recombination. The resulting pVAX1-cGAS (Flag), pVAX1-cGASΔN (Flag), pVAX1-UniSTING (Flag), and pVAX1-IFN-α (Flag) constructs were transformed into DH5α competent cells. Single colonies were cultured and subjected to sequence verification (performed by Comatebio, Changchun, China). Plasmids with a Flag tag facilitated detection, while those without the tag were used for animal experiments. The plasmid containing the GD-Fc sequence maintained in our laboratory was used as a template, and primers were designed to amplify the GD-Fc fragment (GD-Fc-F/R). The PCR product was then inserted into the pVAX1 vector via homologous recombination. The final construct, pVAX1-GD-Fc, was also confirmed by sequencing.

### 2.3. Western Blot

The plasmids were transfected into HEK293T cells cultured in 6-well plates. Two micrograms of plasmid and 6 μL of polyethyleneimine (PEI) were added to 200 μL of DMEM, mixed gently, and incubated at room temperature for 10 min. Cells transfected with the empty pVAX1-Flag plasmid were included as a negative control group. The plasmid-PEI mixtures were added to the HEK293T cells, which were further incubated at 37 °C with 5% CO_2_. After 24 h, the cells were harvested, washed with PBS, and lysed on ice for 10 min using 200 μL of RIPA lysis buffer (supplemented with 1% PMSF) per well. Protein samples were separated by SDS-PAGE and transferred onto PVDF membranes. Following blocking with 5% skim milk, the membranes were incubated with the following primary antibodies: mouse anti-flag antibody (1:10,000; Sigma-Aldrich, St. Louis, MO, USA) or mouse polyclonal anti-GD protein antibody (1:200; kept in the laboratory). After three washes with PBST, the membranes were incubated with DyLight 800-labeled goat anti-mouse IgG (H+L) HSA antibody (1:20,000; KPL, Gaithersburg, MD, USA) or 680RD donkey anti-rabbit IgG antibody (1:10,000; LI-COR, Lincoln, NE, USA) for 1 h at room temperature. After the samples were washed, detection was performed using a Odyssey CLX near-infrared fluorescence scanning imaging system (LI-COR, Lincoln, NE, USA).

### 2.4. Indirect Immunofluorescence Assay

HEK293T cells were seeded into 24-well plates, and 0.5 μg of plasmid was added to 100 μL of DMEM, followed by the addition of 1.5 μL of PEI. Cells transfected with the empty pVAX1-Flag plasmid were included as a negative control group. After incubation, the plasmid-PEI mixtures were then added to HEK293T cells. After 24 h, the supernatant was discarded, and the cells were fixed with 4% paraformaldehyde at room temperature for 15 min, followed by three washes with PBS. The cells were permeabilized with 0.2% Triton X-100 and blocked with 1% BSA at room temperature for 30 min. After the cells were washed, primary antibodies, mouse anti-Flag antibody (diluted 1:1000 in PBS containing 1% BSA) or mouse anti-GD protein monoclonal antibody (diluted 1:1000 in PBS containing 1% BSA) were added and incubated at 37 °C for 1 h, followed by three washes with PBS. The secondary antibody, goat anti-mouse IgG (H+L) Alexa Fluor™ 488 (diluted 1:1000 in PBS; Thermo Fisher, Waltham, MA, USA), was added and the cells were incubated at 37 °C for 1 h. After the cells were washed, DAPI was used to stain the nuclei at 37 °C for 1 h, after which the cells were washed three times with PBS. Finally, the cells were imaged using a fluorescence microscope.

### 2.5. Quantitative PCR Detection of Intracellular mRNA Levels

Total cellular RNA was extracted using a total RNA extraction kit (Bioflux, Hangzhou, China) from cells transfected with the following plasmids: pVAX1-Flag, pVAX1-cGAS-Flag, pVAX1-cGASΔN-Flag, pVAX1-IFN-α-Flag, and pVAX1-UniSTING-Flag plasmids, as well as from cells treated with recombinant IFN-α protein (1000 IU/mL) as a control. The extracted total RNA was reverse transcribed into cDNA, which was subsequently used as a template for quantitative PCR (qPCR) analysis of TBK1, IP10, ISG54, and ISG56 gene expression. The primers used are listed in Table 1. The reaction system was prepared according to the ChamQ Universal SYBR qPCR Master Mix (Vazyme, Nanjing, China) manufacturer’s instructions: 10 μL of 2 × ChamQ Universal SYBR qPCR Master Mix, 0.4 μL of forward primer, 0.4 μL of reverse primer, 1 μL of cDNA, and 8.2 μL of ddH_2_O. After thorough mixing, the samples were subjected to the following qPCR program: initial denaturation at 95 °C for 30 s; 40 cycles of 95 °C for 10 s and 60 °C for 30 s; and a melt curve analysis using the instrument’s default settings. Quantitative PCR was performed using a Q5 instrument (Thermo Fisher, Waltham, MA, USA).

### 2.6. Virus Titration

PK-15 cells were seeded into 96-well plates, and when the cells reached 90% confluence, the viral titers of PRV-GFP and VSV-GFP were determined. Viral supernatants were collected and serially diluted tenfold in DMEM, with eight replicates for each dilution. The diluted virus suspensions were added to the PK-15 cell monolayer and incubated at 37 °C for 1 h. After incubation, the cells were washed three times with PBS, followed by the addition of maintenance medium containing 2% FBS. The cells were cultured at 37 °C with 5% CO_2_ for an additional four days to observe cytopathic effects (CPE). The number of wells exhibiting CPE was recorded, and the TCID_50_ was calculated according to the Reed-Muench method [32].

### 2.7. Immunization and Challenge Procedure for In Vivo Experiments in Mice

A total of 53 five-week-old BALB/c mice were used. Immunization groups (*n* = 8 mice per group) received the following formulations: Group 1 (GD-cGAS): 50 μg pVAX1-GD-Fc and 25 μg pVAX1-cGAS; Group 2 (GD-IFN-α): 50 μg pVAX1-GD-Fc and 25 μg pVAX1-IFN-α; Group 3 (GD-UniSTING): 50 μg pVAX1-GD-Fc and 25 μg pVAX1-UniSTING; Group 4 (GD-DNA): 50 μg pVAX1-GD-Fc; and Group 5 (GD-protein): 30 μg GD-Fc protein stored in our laboratory mixed with an equal volume of Montanide ISA 201 adjuvant [33]. The mice were immunized via intramuscular injection into the hind leg. The remaining 13 mice were immunized with PBS, of which 8 served as the PBS-injected negative control (NC) group and 5 served as the PRV challenge control group (Mock (PRV)). A booster immunization was administered 14 days after the initial immunization, and a third immunization (except in the GD-protein group) was performed at 21 days postvaccination (dpv). One week after the second immunization, with the exception of the challenge control group, three mice per group were bled and euthanized for splenic lymphocyte isolation. The remaining mice were subjected to viral challenge three days after the completion of the immunization regimen. All mice except the negative control group were challenged with PRV-HLJ8 (1.4 × 10^3^ TCID_50_ per mouse) by intramuscular injection into the hind leg at 24 dpv, and mortality and clinical signs were observed within 14 days after challenge.

### 2.8. Clinical Signs and Histopathology

After the challenge, the clinical signs of the mice were recorded daily, with different signs assigned corresponding scores: 0 for no abnormality, 1 for itching or gnawing, 2 for agitation or ruffled fur, 3 for self-mutilation, and 4 for a moribund state or death. For each mouse, only the highest observed score was recorded; for example, if a mouse exhibited both ruffled fur and self-mutilation, a score of 3 was assigned. No further scores were recorded after a mouse died. A clinical sign score curve was plotted on the basis of these scores. Upon disease onset, death, or euthanasia, brain tissue was collected from the mice and fixed in formalin solution. Brain tissues from *n* = 3 mice per group were collected for histopathological evaluation. The fixed tissue was then dehydrated, embedded in paraffin, sectioned, and stained for observation of pathological changes in the brain. Semi-quantitative scoring was based on the severity of lesions and ranged from 0 to 4: a score of 0 indicated no lesion; 1 indicated mild neuronal necrosis/proliferation/atrophy; 2 indicated mild neuronal necrosis/proliferation/atrophy with diffuse infiltration; 3 indicated moderate neuronal necrosis/proliferation/atrophy with diffuse infiltration and neuronophagia; 4 indicated extensive neuronal necrosis/proliferation/atrophy with diffuse infiltration and neuronophagia. Tissue sections were evaluated by veterinary pathologist who was blinded to the group assignments.

### 2.9. Isolation of Splenic Lymphocytes from Mice

After the mice were euthanized by cervical dislocation, the abdominal cavity was opened, and the spleen was removed and washed with sterile PBS. A mouse spleen lymphocyte isolation kit (Haoyang, Tianjin, China) was used for lymphocyte isolation. Briefly, the spleen was cut into pieces and ground, and the resulting single-cell suspension was filtered through a 70 μm cell strainer using homogenization buffer. The cells were collected by centrifugation at 400× *g* for 10 min and resuspended in 1 mL of sample dilution buffer. Separation solution was added to a sterile, siliconized centrifuge tube, and the cell suspension was gently layered on top of the separation solution. The mixture was subsequently centrifuged at 400× *g* and 20 °C for 30 min. The upper dilution layer was discarded, and the second layer, containing lymphocytes, was carefully aspirated and transferred to a new sterile, siliconized centrifuge tube. The cells were subsequently washed with washing buffer and centrifuged at 400× *g* for 10 min. The supernatant was discarded, and the cells were washed twice more with washing buffer and centrifuged at 250× *g* for 10 min each time. Finally, the cells were resuspended in PBS for immediate use or in cell freezing solution and stored at −80 °C.

### 2.10. Phenotypic Characterization of T Cells by Flow Cytometry

Splenic lymphocytes at 21 dpv were washed twice with ice-cold PBS and centrifuged at 500× *g* for 5 min. Then, the cell pellets were resuspended in 100 μL of staining buffer (BD Biosciences, San Jose, CA, USA) mixed with antibodies against rat anti-mouse CD3e-SPRD (SouthernBiotech, Birmingham, AL, USA), rat anti-mouse CD4-FITC (SouthernBiotech, Birmingham, AL, USA) and rat anti-mouse CD8a-PE (SouthernBiotech, Birmingham, AL, USA) on ice for 30 min in the dark. After centrifugation at 500× *g* for 5 min, the cell pellets were washed twice with cold PBS and resuspended in 500 μL of staining buffer. The cell samples were transferred to flow cytometry tubes and loaded onto a flow cytometer (Apogee, Hemel Hempstead, England, UK). Data analysis was performed using FlowJo v10.8.1.

### 2.11. Lymphocyte Proliferation Assay

To detect T cell antigen-specific responses, we cocultured T cells with UV-inactivated PRV. Splenic lymphocytes at 21 dpv were added to a 96-well plate at 1 × 10^5^ cells per well and stimulated with inactivated PRV-HLJ8 (1 × 10^6^ TCID_50_/mL), with six replicate wells for each sample. The 96-well plate was placed in a 5% CO_2_ incubator at 37 °C for 72 h. Then, 10 μL of CCK8 was added per well, and the plate was incubated at 37 °C for 3 h. The OD450 value of the cell plate was measured at 450 nm, and the relative proliferation index was calculated as the ratio of the average OD value of the virus-stimulated wells to the average OD value of the unstimulated wells.

### 2.12. Statistical Analysis

All experiments were performed with three independent replicates, and the error bars indicate the standard deviation (SD). In the viral titer analysis, the differences between means and 95% confidence intervals (CI) were calculated using the Student’s *t* test. Besides, survival analysis was analyzed by log-rank/Mantel–Cox test. Statistically significant differences were analyzed by one-way ANOVA and multiple comparisons using Tukey’s test in GraphPad Prism 8.0 software. A *p*-value < 0.05 was considered to indicate statistical significance.

## 3. Results

### 3.1. Construction and Expression of Plasmids

**Figure 1:**
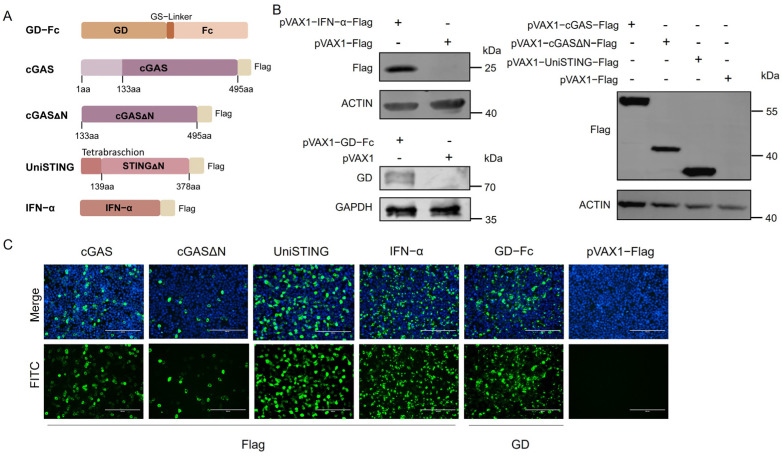
Construction and expression of the GD antigen and adjuvants. (**A**) Schematic diagram of pVAX1-GD-Fc-Flag, pVAX1-cGAS-Flag, pVAX1-cGASΔN-Flag, pVAX1-UniSTING-Flag and pVAX1-IFN-α-Flag. (**B**) Detection of GD-Fc, cGAS, cGASΔN, UniSTING, and IFN-α protein expression in HEK 293T cells by Western blotting with anti-PRV GD polyclonal antibody and anti-Flag antibodies. Antibodies against housekeeping proteins included mouse anti-β-actin, rabbit anti-β-actin, and mouse anti-GAPDH. DyLight 800-labeled goat anti-mouse IgG (H+L) HSA antibody and 680RD donkey anti-rabbit IgG antibody were utilized as secondary antibodies. (**C**) IFA of GD-Fc, cGAS, cGASΔN, UniSTING, and IFN-α expression. Proteins were detected using a mouse anti-Flag monoclonal antibody and an anti-PRV GD monoclonal antibody as primary antibodies, followed by a goat anti-mouse IgG (H+L) Alexa Fluor 488 secondary antibody. The nuclei were counterstained with DAPI. All the images were acquired under identical fluorescence parameters. Scale bar: 200 μm.

Sequencing analysis confirmed the successful construction of all the plasmids, including pVAX1-cGAS (Flag), pVAX1-cGASΔN (Flag), pVAX1-UniSTING (Flag), pVAX1-IFN-α (Flag), and pVAX1-GD-Fc. The sequences of cGAS, cGASΔN, UniSTING, and IFN-α were successfully cloned and inserted into the pVAX1 vector and tagged with flag for detection. The GD gene was fused with an Fc sequence and connected via a GS-Linker, and this construct was also successfully cloned and inserted into the pVAX1 vector. A schematic diagram of the plasmid construction is shown in Figure 1A. To verify the in vitro expression, the plasmids were transfected into HEK-293T cells. Western blot (Figure 1B) and indirect immunofluorescence assays (Figure 1C) revealed that the cGAS, cGASΔN, UniSTING, IFN-α and GD-Fc proteins were expressed.

### 3.2. A cGAS, cGASΔN, IFN-α, and UniSTING Exhibit Effective Antiviral Activity In Vitro

**Figure 2:**
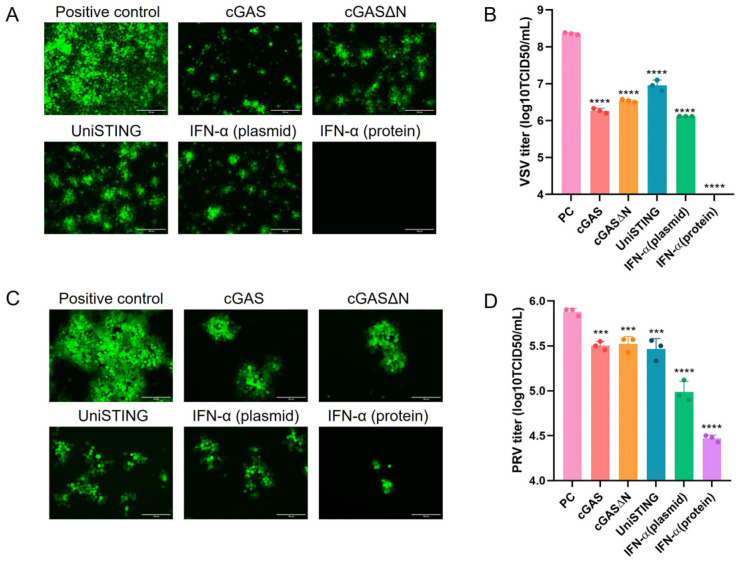
Antiviral effects of cGAS, cGASΔN, UniSTING and IFN-α in PK-15 cells. After the PK-15 cells were treated for 24 h with pVAX1-Flag, pVAX1-cGAS-Flag, pVAX1-cGASΔN-Flag, pVAX1-UniSTING-Flag, pVAX1-IFN-α-Flag and recombinant IFN-α, the cells were infected with VSV-GFP (MOI = 0.001) or PRV-GFP (MOI = 0.01) for an additional 24 h. The expression of GFP was detected using a fluorescence microscope indicating that the cells were infected with VSV-GFP (**A**) or PRV-GFP (**C**); scale bar: 150 μm. The cell supernatants were collected, and the viral titers of VSV-GFP (**B**) and PRV-GFP (**D**) were determined in PK-15 cells. The data are presented as the mean ± SD from three independent experiments. In the viral titer analysis, the differences between means and 95% CI were calculated using the Student’s *t* test. *p* values were calculated using one-way ANOVA, followed by multiple comparisons with Tukey’s test as *p* < 0.001 (***) and *p* < 0.0001 (****).

To investigate whether the proteins cGAS, cGASΔN, IFN-α, and UniSTING have antiviral effects in PK-15 cells. The pVAX1-cGAS-Flag, pVAX1-cGASΔN-Flag, pVAX1-IFN-α-Flag, and pVAX1-UniSTING-Flag plasmids were transfected into PK-15 cells, and recombinant IFN-α protein (1000 IU/mL) was used as a control. At 24 h after transfection, each well was infected with 100 TCID_50_ of VSV-GFP (MOI = 0.001). The results revealed that the cGAS, cGASΔN, IFN-α, and UniSTING plasmids partially inhibited VSV-GFP replication (Figure 2A), whereas recombinant IFN-α completely suppressed VSV-GFP replication. Virus-containing supernatants were collected for virus titration, and compared with those of the virus-infected control group. Viral titers were log_10_-transformed prior to analysis, and geometric mean fold change (GMFC) with 95% CI were calculated for each group compare to the control. As shown in Figure 2B, all experimental groups demonstrated a statistically significant reduction in viral titer (*p* < 0.0001). The most effective inhibition was observed in the cGAS and IFN-α plasmid groups, with 175-fold (GMFC = 0.0081; 95% CI: 0.0040 to 0.0164) and 123-fold reductions (GMFC = 0.0057; 95% CI: 0.0044 to 0.0074), respectively. The cGASΔN and UniSTING groups also exhibited significant reductions of 66-fold (GMFC = 0.0150; 95% CI: 0.0097 to 0.0233) and 25-fold (GMFC = 0.0392; 95% CI: 0.0089 to 0.1723) (Figure 2B). These results indicated that the cGAS, cGASΔN, IFN-α, and UniSTING plasmids confer varying degrees of antiviral activity against VSV replication in PK-15 cells. Afterward, we verified the inhibitory effects of cGAS, cGASΔN, IFN-α, and UniSTING on PRV (MOI = 0.01). Using the same methodology as for VSV-GFP, the results revealed that cGAS, cGASΔN, IFN-α, and UniSTING could also inhibit PRV-GFP replication (Figure 2C). Compared with those in the PRV-GFP infection control group, the viral titers in the groups in which cGAS, cGASΔN, and UniSTING were present were significantly lower (*p* < 0.001), whereas the viral titer in the IFN-α plasmid group was extremely low (*p* < 0.0001) (Figure 2D). However, the recombinant IFN-α protein did not completely suppress PRV-GFP expression (*p* < 0.0001) (Figure 2C,D). These results indicated that plasmid-expressed cGAS, cGASΔN, IFN-α, and UniSTING proteins inhibited PRV replication to varying degrees in PK-15 cells and that the IFN-α group displayed the most pronounced inhibitory effect.

**Figure 3:**
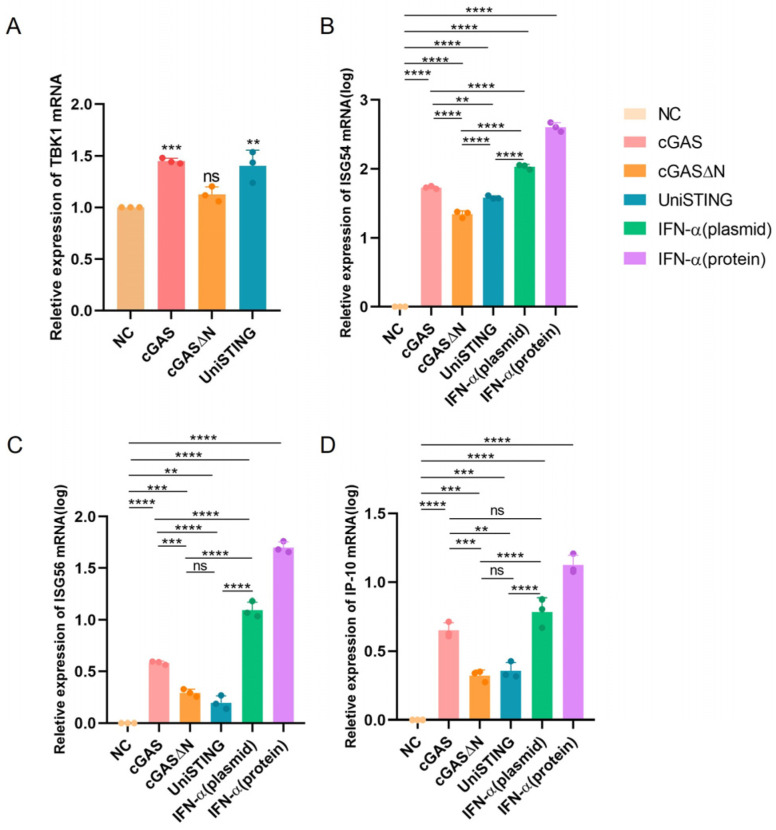
Effects of cGAS, cGASΔN, UniSTING and IFN-α on the cGAS-STING signaling pathway and interferon-stimulated gene mRNA levels. PK-15 cells were treated with pVAX1-Flag, pVAX1-cGAS-Flag, pVAX1-cGASΔN-Flag and pVAX1-UniSTING-Flag for 12 h, after which the mRNA level of TBK1 was detected (**A**). The mRNA levels of ISG54 (**B**), IP-10 (**C**) and ISG56 (**D**) were detected after treatment with pVAX1-Flag, pVAX1-cGAS-Flag, pVAX1-cGASΔN-Flag, pVAX1-UniSTING-Flag, pVAX1-IFN-α-Flag and recombinant IFN-α for 24 h. Data are presented as the mean ± SD from three independent experiments. *p* values were calculated using one-way ANOVA followed by multiple comparisons using Tukey’s test as follows: *p* < 0.01 (**), *p* < 0.001 (***), *p* < 0.0001 (****), and ns: not significant.

We hypothesized that their antiviral activity might be mediated through IFN-I-induced JAK-STAT pathway activation. pVAX1-cGAS-Flag, pVAX1-cGASΔN-Flag, pVAX1-UniSTING-Flag, and pVAX1-IFN-α-Flag plasmids were transfected into PK-15 cells, while recombinant IFN-α protein was used as a positive control. The mRNA levels of TBK1, ISG54, ISG56 and IP10 were determined 24 h after transfection. Compared with those in the negative control (NC) group, TBK1 mRNA levels were significantly elevated in cells expressing cGAS (*p* < 0.001) and UniSTING (*p* < 0.01), whereas no significant change was observed in the cGASΔN group (Figure 3A). Previous studies have shown that the sensitivity of cGASΔN to self-DNA varies across species [26], and we speculated that the sensitivity of the porcine cGASΔN variant to self-DNA may be relatively low. ISG54 and ISG56 are important antiviral interferon-stimulated genes involved in antiviral immune responses. Compared with the NC group, transfection with plasmids encoding cGAS, cGASΔN, UniSTING, and IFN-α, as well as treatment with recombinant IFN-α protein, significantly increased the mRNA expression of ISG54 and ISG56 in PK-15 cells (*p* < 0.01, *p* < 0.0001) (Figure 3B,C). IP-10 is a key immunoregulatory chemokine that plays a critical role in antiviral responses, and its production is regulated primarily by the cGAS-STING pathway. Both recombinant IFN-α protein expression and transfection with the cGAS, cGASΔN, UniSTING, and IFN-α plasmids markedly increased IP-10 mRNA expression levels (*p* < 0.001, *p* < 0.0001) (Figure 3D). These results demonstrated that plasmids expressing cGAS, cGASΔN, UniSTING, and IFN-α significantly increased the expression of ISG54, ISG56, and IP-10, thereby exerting antiviral immune effects. Notably, the induction of these three ISGs by cGAS was significantly greater than that induced by cGASΔN and UniSTING (*p* < 0.01). The recombinant IFN-α protein exhibited the most pronounced effect on the expression of the aforementioned ISGs, which is consistent with its strong antiviral activity at the cellular level. Furthermore, we hypothesized that the sensitivity of the porcine cGASΔN variant to self-DNA may be reduced, which partially compromises its ability to activate ISGs.

### 3.3. cGAS or IFN-α as an Adjuvant Enhances the CD8+ T Cell-Specific Immune Response

**Figure 4:**
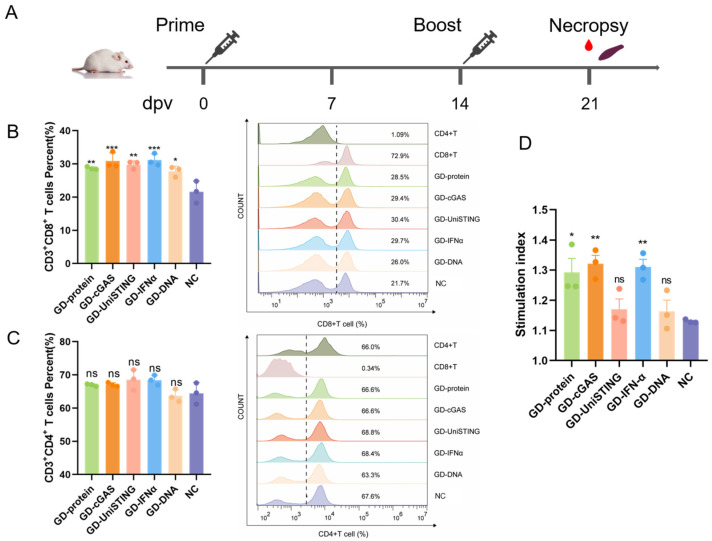
Immunogenicity of DNA vaccines in mice. (**A**) Immunization procedures for mice. The mice were immunized at 0 and 14 days. Spleens and blood were collected at 21 days. (**B**,**C**) The proportions of CD3+CD4+ T cells and CD3+CD8+ T cells were detected by flow cytometry in spleen lymphocytes at 21 dpv. Data analysis was performed using FlowJo v10.8.1. (**D**) PRV lymphocyte proliferation assay. The spleen lymphocytes were stimulated with inactivated PRV-HLJ8 for 48 h, and the average OD value of the antigen-stimulated wells was compared to that of the unstimulated wells. The data are presented as the mean ± SD (*n* = 3). *p* values were calculated using one-way ANOVA followed by multiple comparisons with Tukey’s test as follows: *p* < 0.05 (*), *p* < 0.01 (**), *p* < 0.001 (***), and ns: not significant.

On the basis of the aforementioned data, we speculated that compared with cGASΔN, porcine full-length cGAS is more sensitive to self-DNA and is a better immunostimulatory adjuvant. Therefore, we further studied the potential of wild-type cGAS as an immunostimulatory adjuvant in vivo. To evaluate whether cGAS/IFN-α/UniSTING enhances the level of antigen-specific acquired immune responses, mice were immunized with pVAX1-GD-Fc mixed with pVAX1-cGAS, pVAX1-IFN-α, or pVAX1-UniSTING. The pVAX1-GD-Fc plasmid or GD-Fc protein served as the control, with an additional PBS group used as a negative control. The animal experiment schedule is shown in Figure 4A, in which two immunizations were administered on days 0 and 14; one week after the second immunization, the splenic lymphocytes were harvested. Flow cytometry was used to assess the proportions of CD3+CD4+ T cells and CD3+CD8+ T cells. The analysis was performed with FlowJo v10.8.1, and the gating strategy and flow cytometry dot plots are provided in Figure S1. The percentage of CD3+CD4+ T cells was not significantly different between the vaccine-immunized group and the control group, whereas the percentage of CD3+CD8+ T cells was significantly greater (*p* < 0.05, *p* < 0.01, *p* < 0.001) in the vaccine-immunized group than in the NC group that received the PBS alone (Figure 4B,C). These findings suggested that the vaccine-induced immune response was mediated primarily through the stimulation of CD8+ T cells. To detect T cell antigen-specific responses, we cocultured T cells with UV-inactivated PRV. T lymphocyte proliferation was evaluated using a CCK-8 assay. Compared with that in the NC group, the proliferation of T lymphocytes in the GD-Fc protein vaccine group (*p* < 0.05), cGAS plasmid group (*p* < 0.01), and IFN-α plasmid group (*p* < 0.01) significantly increased, whereas no significant differences were noted for the UniSTING plasmid group or the GD-Fc plasmid group (Figure 4D). These results demonstrated that the adjuvants cGAS and IFN-α significantly enhanced CD8+ T cell immune responses in mice.

### 3.4. pVAX1-GD-Fc Combined with cGAS, IFN-α, or UniSTING Provides Early Protection Against PRV Challenge in Mice

**Figure 5:**
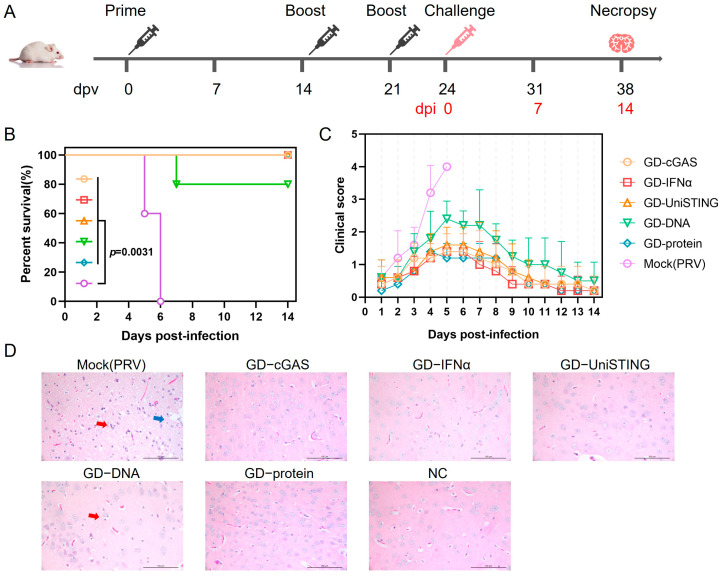
The PRV gD DNA vaccine with cGAS, UniSTING, and IFN-α protects mice from PRV challenge. (**A**) Immunization and challenge schedule in mice. The mice were immunized at 0, 14, and 21 days, challenged with PRV-HLJ8 at 24 days after immunization, and sacrificed at 14 days post-challenge. Black font indicates days postvaccination (dpv), and red font indicates days postinfection (dpi) (**B**) Survival curve of mice at 14 days post-challenge. (**C**) The clinical signs of the mice were recorded after challenge, with different signs assigned corresponding scores: 0 for no abnormality, 1 for itching or gnawing, 2 for agitation or ruffled fur, 3 for self-mutilation, and 4 for a moribund state or death. (**D**) Hematoxylin-eosin (HE) staining of brain sections. The blue arrow points to neuronal necrosis; the red arrow points to neuronophagia. Scale bar: 100 μm. The data are presented as the mean ± SD (*n* = 5). The survival analysis was analyzed by log-rank/Mantel–Cox test.

To verify whether cGAS/IFN-α/UniSTING can enhance the in vivo protective efficacy of the antigen, mice were immunized with a mixture of pVAX1-GD-Fc combined with pVAX1-cGAS, pVAX1-IFN-α, or pVAX1-UniSTING. Mice immunized solely with the pVAX1-GD-Fc plasmid served as the control group, while mice immunized with the GD-Fc protein with ISA201 adjuvant served as the positive control. Additionally, two more groups were included: a mock-challenged group (Mock (PRV)), which PBS-injected and was subsequently challenged by PRV, and a negative control group (NC), which PBS-injected without challenge. The mice were challenged with PRV-HLJ8 at 24 dpv (Figure 5A). The survival of the mice was monitored for 14 days postinfection (dpi) to generate survival curves (Figure 5B). The mock-challenged group (*n* = 5) had a median survival of 6 days. In contrast, the median survival was not reached in any of the vaccinated groups (*n* = 5 per group) by the end of the observation period. This includes the group immunized with the pVAX1-GD-Fc plasmid alone, despite a single death on day 7. The log-rank test confirmed that each vaccinated group showed a statistically significant survival benefit compared to the control group (χ^2^ = 8.731, df = 1, *p* = 0.0031). Clinical sign scoring further demonstrated that, compared with the group receiving only the pVAX1-GD-Fc plasmid, the groups with the mixed adjuvants exhibited lower clinical sign scores (Figure 5C), with no significant differences observed among the three mixed adjuvant groups. The surviving mice at 21 dpi and moribund mice were euthanized, followed by histopathological examination. Brain sections were stained with hematoxylin-eosin (HE) to observe histopathological changes. In the mock challenge group, neuronal necrosis and neuronophagia were observed in the brain sections, which are typical neurotropic changes caused by PRV [34]. The pVAX1-GD-Fc plasmid group also exhibited neuronophagia. Notably, the brains of the NC, cGAS, IFN-α, and UniSTING groups were normal, and no neuronal necrosis or neuronophagia were observed (Figure 5D). In addition, semi-quantitative scoring was performed, with GD-DNA vaccination and PRV challenge showing the highest score of 2 ± 1 (Figure S2). These results indicated that a PRV gD-based DNA vaccine candidate adjuvanted with cGAS, UniSTING, or IFN-α enhanced protective immunity in mice.

## 4. Discussion

The cGAS-STING signaling axis represents a highly promising class of molecular adjuvants for vaccine development. In this study, we evaluated the adjuvant potential of cGAS, UniSTING, and IFN-α when they were coadministered with a PRV gD-based DNA vaccine in mice. Notably, in previous studies, to achieve the best protection against infection, infection experiments were usually performed 2 weeks or later after immunization, when antibody titers reached relatively high levels [4,5]. In contrast, our study demonstrated that the present formulation enabled 100% survival in mice challenged with lethal PRV as early as 3 days after completion of the immunization protocol. These findings demonstrate that the use of cGAS, UniSTING, and IFN-α as immune-stimulating adjuvants can significantly increase early immune activation, thereby rapidly inducing an immune response and substantially advancing the onset of vaccine-mediated protection. This approach may effectively narrow the immunological gap in practical applications. Furthermore, prior studies have investigated the protective efficacy of both an mRNA vaccine encoding the PRV gD gene and a DNA vaccine expressing the PRV gD protein via the recombinant plasmid pVAX-gD. Both the mRNA and pVAX-gD plasmids induced high levels of neutralizing antibodies and antigen-specific B and T cell responses in mice. When challenged with the PRV-XJ strain at 8 weeks after primary immunization, 90% of mRNA-vaccinated mice survived, whereas 100% of DNA-vaccinated mice survived [35]. In the present study, mice immunized with the pVAX1-GD-Fc plasmid alone showed only 80% protection. This discrepancy may be attributed to differences in the pathogenicity of the viral strains used—the PRV HLJ8 strain employed here caused more than 90% mortality in mice even at a challenge dose of 1.4 × 10^3^ TCID_50_. Notably, all the mice receiving the DNA vaccine supplemented with immunostimulatory adjuvants survived, indicating that these adjuvants significantly enhanced protection against PRV HLJ8 challenge. However, in our study only a neutralizing antibody titer of 1:16 was detected in the GD-Fc protein group one week after the second immunization, while the DNA vaccine group produced nearly no neutralizing antibodies (Table S1), which contrasts sharply with previous reports [35,36]. Earlier work demonstrated that immunization with the pVAX-gD plasmid resulted in peak neutralizing antibody titers (20) at 4 weeks after primary immunization, followed by a gradual decline [35]. A recent study on a self-amplifying mRNA vaccine encoding the PRV gD protein reported a neutralizing antibody titer of 1:128.5 at 42 days after immunization [37]. Nevertheless, in our study, the DNA vaccine still provided significant protection, as evidenced by an 80% survival rate in mice immunized with the pVAX1-GD-Fc plasmid alone. These findings suggest that the DNA vaccine in this study may confer protection primarily through eliciting cellular immune responses.

cGASΔN variants from different species exhibit varying sensitivities to self-DNA and viral DNA. In the case of porcine-derived cGAS, compared with truncated cGASΔN, the full-length protein induces IFN-I production in cells more effectively, which is consistent with the findings of previous studies [38]. Notably, cGASΔN also exhibited antiviral activity in cellular infection models. We hypothesized that during viral infection, both viral DNA and host-derived DNA (released because of virus-induced cellular damage) concurrently activate the cGAS-STING pathway, triggering downstream signaling and IFN-α production via both wild-type cGAS and cGASΔN. However, compared with wild-type cGAS, cGASΔN demonstrated inferior antiviral activity and IFN-α induction capacity. We hypothesize that the sensitivity of the porcine cGASΔN variant to self-DNA is relatively low. Intratumoral or systemic delivery of UniSTING mRNA via lipid nanoparticles (LNP) can effectively activate the cGAS-STING pathway, promote dendritic cell (DC) maturation, and elicit antigen-specific CD8+ T cell immune responses [26]. In the present study, although GD-UniSTING immunization significantly increased the percentage of CD3+CD8+ T cells, the proliferative capacity of antigen-specific T lymphocytes remained relatively limited. This phenomenon may be attributed to the low in vivo transfection efficiency of intramuscularly administered DNA vaccines, resulting in an insufficient dosage of the UniSTING agonist, which fails to fully activate T cells and thereby constrains the intensity of the response. Notably, despite the suboptimal T cell activation, GD-UniSTING immunization provided 100% protection in the challenge experiment, significantly outperforming the 80% protection observed in the GD-DNA vaccine group. These findings suggest that IFN-I and ISGs induced by UniSTING play crucial roles in conferring protection against PRV infection. However, sustained or excessive IFN-I production and inflammatory responses may conversely inhibit T cell function [39]. On the other hand, if the DNA vaccine is taken up by nonprofessional antigen-presenting cells, the expressed antigen and UniSTING may fail to efficiently activate DCs, leading to suboptimal cross-presentation. Therefore, employing appropriate delivery vehicles in DNA vaccine design could enable targeted codelivery of antigens and immune agonists, which would not only increase antigen uptake and promote antigen-presenting cells (APCs) maturation but also increase T cell activation and proliferation, ultimately improving overall vaccine-induced protective immunity.

Compared with cGAS and UniSTING, IFN-α had a stronger antiviral effect on cells. However, IFN-α has faced significant barriers to clinical adjuvant application because of its short half-life and narrow therapeutic window. Many studies have recently used IFN-α as an adjuvant to enhance the protective effect of vaccines. For example, the use of IFN-α and IFN-λ as adjuvants after influenza B vaccination or viral infection in ferrets can enhance their immune effects [40]. However, systemic exposure to IFN-α can trigger severe inflammatory responses and unintended immunosuppressive effects, posing safety concerns. Targeted delivery to specific immune organs or tissues may help mitigate these potential risks. One study developed a subunit vaccine by genetically fusing IFN-α, the SARS-CoV-2 RBD, and an Fc fragment (I-R-F). This design induced high levels of long-lasting neutralizing antibodies and provided protection even as a single-dose vaccine without adjuvants [41]. The Fc portion enables the vaccine to target APCs expressing Fc receptors, thereby enhancing the accumulation of the antigen and IFN-α in the draining lymph nodes while reducing side effects. The clinical application of IFN-α as a vaccine adjuvant faces significant challenges because of its short half-life and narrow therapeutic window. The use of an Fc fragment can extend the protein’s half-life while reducing its likelihood of being transported to lysosomes for degradation [42]. Thus, the fusion of IFN-α with an Fc fragment represents an effective strategy to prolong its half-life. Additionally, many mRNA-LNP platforms tend to be enriched in immune organs [43]. Therefore, delivering IFN-α in mRNA form as an adjuvant may represent another promising strategy.

In terms of antiviral activity, cGAS, UniSTING, and IFN-α have inhibitory effects on the replication of VSV in cells, whereas their ability to inhibit PRV is weaker. This difference may be attributed to the greater sensitivity of VSV to IFN-α, whereas PRV employs multiple strategies to counteract IFN-I production [19]. On the other hand, cGAS, UniSTING, and IFN-α function primarily to enhance immune responses and can serve as adjuvants in mice. Compared with that in the pVAX1-GD-Fc plasmid group without adjuvant, the protective effect was improved with adjuvant. Thus, the DNA vaccine supplemented with adjuvants such as cGAS, UniSTING, or IFN-α may enhance protection primarily through stimulation of cellular immunity. However, further experiments are necessary to fully elucidate these mechanisms. In summary, in this study, mice were immunized with cGAS, UniSTING, and IFN-α as adjuvants in the PRV gD DNA vaccine, which enhanced the protective efficacy of the gD DNA vaccine against PRV challenge, thereby conferring resistance to PRV challenge. These results demonstrate that these agents serve as immunostimulatory agents capable of enhancing the protective efficacy of DNA vaccines. To further explore their adjuvant potential for veterinary vaccine applications, subsequent studies should include immunization experiments in swine models to validate their immunoenhancing effects.

## Figures and Tables

**Table 1:** Primer sequences.

Name	Sequence
cGAS-F1	TAGGGAGACCCAAGCTGGCTAGCATGGCGGCCCGGCGGGGA
cGAS-R1	GATCCGAGCTCGGTACCAAGCTTTTACCAAAAAACTGGAAATCCAT
cGASΔN-F	TAGGGAGACCCAAGCTGGCTAGCGCCACCATGCCCGGCGCCTGGAAG
cGASΔN-R	CCGAGCTCGGTACCAAGCTTTTACCAAAAAACTGGAAATCCA
Tebra-F	TAGGGAGACCCAAGCTGGCTAGCATGGGCATTATCAACGAGA
Tebra-R	GAGACTTCAGCTGGGGCCAGGCCGGTAC
STINGΔN-F	CTGGCCCCAGCTGAAGTCTC
STINGΔN-R	ATCCGAGCTCGGTACCAAGCTTTTAGAAGATATCTGAGCGGA
IFN-α-F	TAGGGAGACCCAAGCTGGCTAGCGCCACCATGGATGCCATG
IFN-α-R	ATCCGAGCTCGGTACCAAGCTTTTACTCCTTCTTGCGCAG
GD-Fc-F	GGGAGACCCAAGCTGGCTAGCGCCACCATGGAGACAGACAC
GD-Fc-R	ATCCGAGCTCGGTACCAAGCTTTCACTTCCCCTGAGTCTTAGA
TBK1-Forward	GCCAAGGAGCTACTGCAAAT
TBK1-Reverse	ACATCCACTGGACGAAGGAAG
IP10-Forward	ATAAGGATGGGCCGGAGAGA
IP10-Reverse	GTGGGAGCAGCTAACTTGGT
ISG54-Forward	GCACAGCAATCATGAGTGAGAC
ISG54-Reverse	CTGGCCCCTGCAGTCTTTTA
ISG56-Forward	TCCGACACGCAGTCAAGTTT
ISG56-Reverse	TGTAGCAAAGCCCTGTCTGG
GAPDH-Forward	CCTTCCGTGTCCCTACTGCCAAC
GAPDH-Reverse	GACGCCTGCTTCACCACCTTCT

## Data Availability

The original contributions presented in this study are included in the article/supplementary material. Further inquiries can be directed to the corresponding authors.

## Supplementary Materials

The following supporting information can be downloaded at: https://www.mdpi.com/article/10.3390/pathogens14101026/s1, Figure S1: Flow cytometry analysis.; Figure S2: Semi-quantitative scoring of histopathology of brain. Table S1: Neutralizing antibody titers in serum of each group at 21 dpv.

## Abbreviations

The following abbreviations are used in this manuscript:

PRV	Pseudorabies virus
cGAS	The cyclic GMP-GAP synthase
STING	stimulator of interferon genes
VSV	vesicular stomatitis virus
gD	glycoprotein D
IFN	Interferon
IRF3	Interferon Regulatory Factor 3
TBK1	TANK-Binding Kinase 1
CDNs	cyclic dinucleotides
ISG	interferon stimulated gene
UniSTING	Universal STING complex mimic
PEI	polyethyleneimine
CPE	cytopathic effects
dpv	days post-vaccination
IFA	indirect immunofluorescence assay
NC	Negative Control
SD	standard deviations
HE	Hematoxylin-eosin
