# Supplementary material for: PRV gD-Based DNA Vaccine Candidates Adjuvanted with cGAS, UniSTING, or IFN-α Enhance Protective Immunity
Source: Pathogens. 2025 Oct 11;14(10):1026. doi: 10.3390/pathogens14101026 (PMC12567548; doi:10.3390/pathogens14101026)
Supplement: Supplementary file 1 [file pathogens-14-01026-s001.zip › pathogens-3835664-supplementary.pdf]

Figure S1

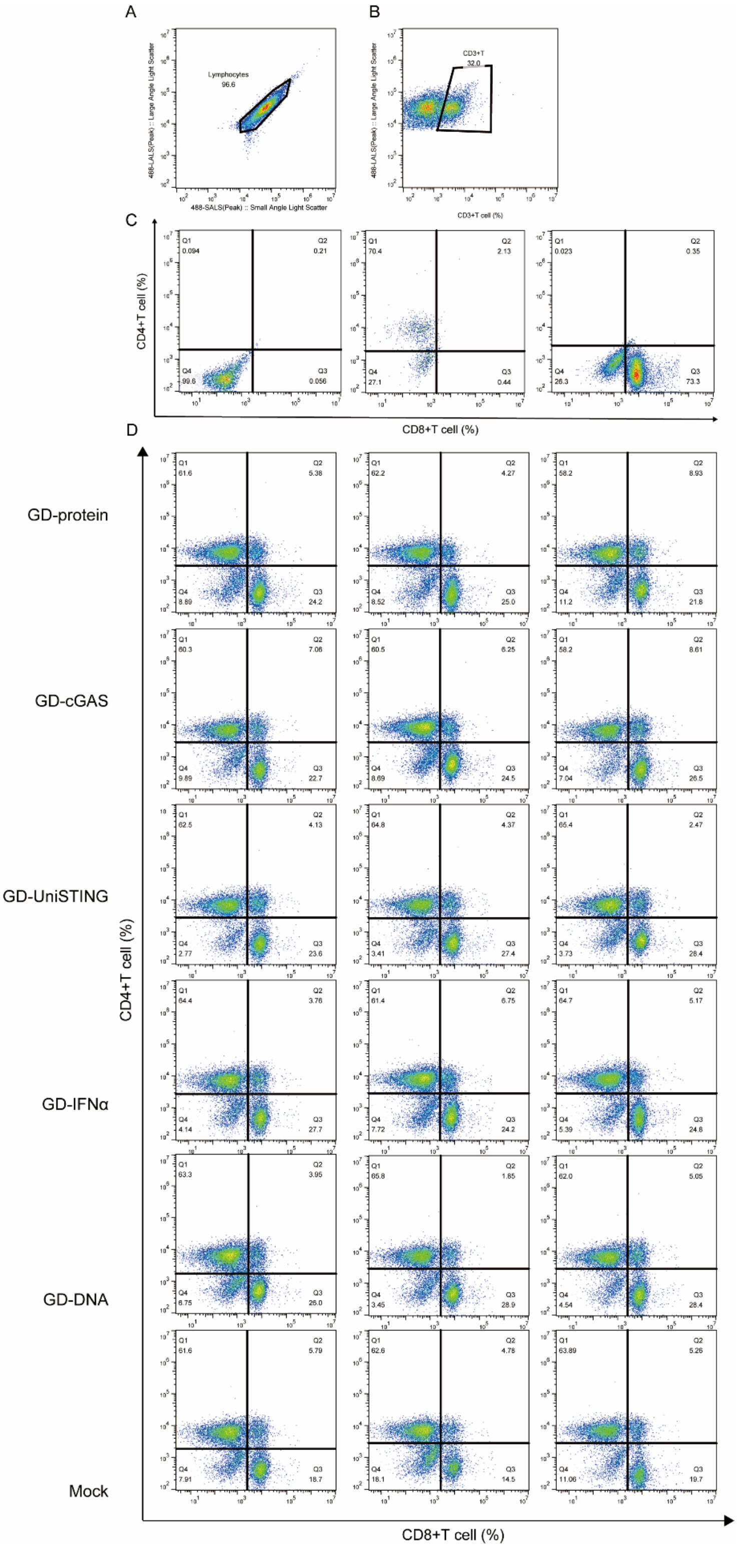

Figure S1. Flow cytometry analysis. (A) Gating strategy for lymphocyte population (using CD3+ T cell single-stain control as an example). (B) Gating strategy for CD3+ T cell population. (C) Flow cytometry plots of single-stain controls for CD3+, CD4+, and CD8+ T cells. (D) Flow cytometry plots of samples from GD-protein, GD-cGAS, GD-UniSTING, GD-IFN-α, GD-DNA, and Mock groups.

Figure S2

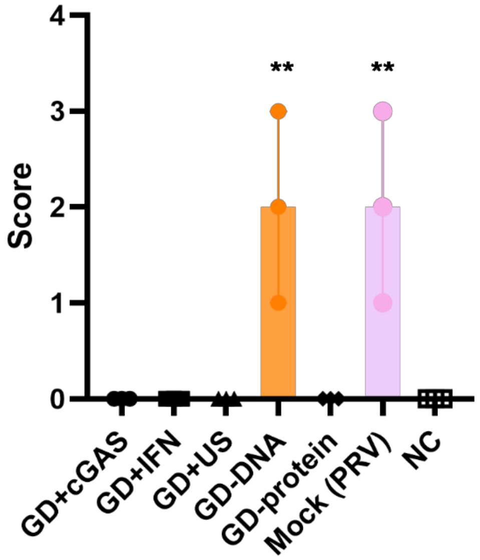

Figure S2. Semi-quantitative scoring of histopathology of brain. Brain sections were stained with hematoxylin-eosin (HE) to observe histopathological changes. Semi-quantitative scoring was based on the severity of lesions and ranged from 0 to 4: a score of 0 indicated no lesion; 1 indicated mild neuronal necrosis/proliferation/atrophy; 2 indicated mild neuronal necrosis/proliferation/atrophy with diffuse infiltration; 3 indicated moderate neuronal necrosis/proliferation/atrophy with diffuse infiltration and neuronophagia; 4 indicated extensive neuronal necrosis/proliferation/atrophy with diffuse infiltration and neuronophagia. The pathological changes in each group were evaluated based on the severity observed in the histopathological sections. The data are presented as the mean±SD from three independent mice. *p* values were calculated using one-way ANOVA, followed by multiple comparisons with Tukey's test as $p<0.01$ (**).

Table S1. Neutralizing antibody titers in serum of each group at 21 dpv

Groups	GD-protein	GD-cGAS	GD-UniSTING	GD-IFN-α	GD-DNA
Neutralizing antibody titers	1:16	<2	<2	<2	<2
